# Important questions in drug allergy and hypersensitivity: consensus papers from the 2018 AAAAI/WAO international drug allergy symposium

**DOI:** 10.1186/s40413-018-0224-1

**Published:** 2018-12-19

**Authors:** Pascal Demoly, Mariana Castells

**Affiliations:** 1 Department of Pulmonology, Division of Allergy, Hôpital Arnaud de Villeneuve, University Hospital of Montpellier, Univ Montpellier and Equipe EPAR, IPLESP, INSERM Sorbonne Université, Paris, France; 2 Department of Medicine, Division of Rheumatology, Immunology and Allergy, Brigham and Women’s Hospital, Harvard Medical School, 60 Fenwood Road, Room 5002N Hale BTM Building, Boston, MA 02115 USA; 3 Drug Hypersensitivity and Desensitizations Center, Brigham and Women’s Hospital, Harvard Medical School, 60 Fenwood Road, Room 5002N Hale BTM Building, Boston, MA 02115 USA; 4 Mastocytosis Center, Brigham and Women’s Hospital, Harvard Medical School, 60 Fenwood Road, Room 5002N Hale BTM Building, Boston, MA 02115 USA

**Keywords:** Drug allergy, Hypersensitivity, Beta-lactam, Radiographic contrast media, In vitro allergy testing, Delayed drug reactions

## Abstract

This article is one of a series of international consensus documents developed from the International Drug Allergy Symposium held at the Joint Congress of the American Academy of Allergy, Asthma & Immunology/World Allergy Organization on March 1, 2018, in Orlando, Florida, USA. The symposium was sponsored by The Journal of Allergy and Clinical Immunology, The Journal of Allergy and Clinical Immunology: *In Practice*, and The World Allergy Organization Journal and chaired by Mariana Castells, MD, PhD, and Pascal Demoly, MD, PhD.

## Unresolved questions in drug hypersensitivity reactions

The prevalence of self-reported drug allergy has been reported up to 8% in the general population [1] and that of beta-lactam up to 15% of hospitalized patients [2]. It varies according to age, drug classes, countries and drug prescription habits. Beyond the variation in prevalence to explain some unresolved questions are the differences in the diagnosis and management which exist from country to country and within the same country [3, 4]. Regardless of the mechanisms, drug allergy and hypersensitivity reactions (DHRs) are a daily worry for clinicians and patients. Although urticaria and maculopapular eruptions are the most frequent manifestations of DHRs, there are many clinical presentations that are life-threatening, require or prolong hospitalization, and entail changes in drug prescription [4]. Both under-diagnosis (due to under-reporting) and over-diagnosis (due to the over-use of the term «allergy») are potential problems [1, 5]. Drug allergy mislabelling can impact individual treatment choices and can lead to the use of more harmful, less effective and more expensive drugs or to the potential reintroduction of a causal drug with severe relapse of DHRs.

DHRs have a significant impact on clinical practice, socio-economy, drug development, and public health. Although tremendous progress has been made over the past two decades in the understanding of the mechanisms of drug allergy and hypersensitivity and their management, several unmet needs remain. Several task forces with representatives from the key stakeholders (research clinicians, regulatory scientists and immuno-toxicologists) have identified the critical data gaps and opportunities and made recommendations on how to overcome some of the barriers to DHRs research and address research needs [4, 6, 7]. What are the key questions and what has been done since the latest task force? A large number of reactions are presumed to be drug-related and of allergic nature, but closer examination reveals that they are not [1, 4, 5]. The diagnosis of DHRs relies on clinical history, skin testing, validated in vitro tests and drug provocation tests [4]. Standardised diagnostic procedures have been published, including those of the European Network of Drug Allergy and the USA Practice Parameters, which have provided consensuses for the diagnosis of specific drugs reactions (reviewed in 4). Validation of clinical tests for all drugs does not exist, and multicentre studies around the world are needed to achieve it. Establishing standard operating procedures and cut-off concentrations for skin tests for most drugs is achievable. The diagnosis of severe cutaneous reactions, including those affecting multi organ systems, remains difficult due to the lack of diagnostic tools. The development of new in vitro biological and genetic testing and in vivo skin testing is crucial for those cases where drug provocation is not possible. The establishment of multi-national, adequately resourced large DHR databases would enable all cases to be collected, which would in turn facilitate epidemiologic, risk factor and pharmacovigilance analysis. Critically, it would address the mechanisms of DHRs and provide the description of phenotypes, their underlying endotypes, and associated biomarkers. However, heterogeneity in practices exist around the world, even in the same country, and standardization is required before outcomes can be measured and understood.

Epidemiologic risk factors for DHRs are not well characterized and may be influenced by regional/national differences in drug prescriptions. All drugs can induce DHRs, but the incidence and risk factors for individual drugs have been poorly defined. The development of a network that can increase the population size from which to capture data on DHRs would be a major advance. The establishment of such a network would need the concomitant development of unique DHR databases collecting standardised data, the nature of which would have to be defined on the basis of expert consensus. The development of such a database would have huge benefits in validating diagnostic procedures and defining the risk factors associated with DHRs. It would permit personalized analysis at the individual level and the country level (related to individual prescribing habits), and would allow longitudinal assessment of the safety of new drugs. It would overcome the major limitation of spontaneous reporting, i.e. under-reporting, by engaging interested clinicians involved in the network. It would assess the socio-economic impact of DHRs, drug allergy pathways, and allow follow-up studies (the natural history of most DHRs is not known).

One of the major reason why we are still lacking reliable in vitro tests is due to a lag in the understanding of the mechanisms of most DHRs. Drugs are capable of inducing all the types of immunological reactions described by Gell and Coombs, with the most common being IgE and T-cell mediated reactions [8]. Drugs can directly interact with the immune system and inflammatory cells, but these interactions do not necessarily lead to clinical symptoms. Not all drugs need to bind covalently to the major histocompatibility complex in order to induce an immune response. There is speculation that some drugs, without undergoing the classical antigen processing and presentation pathway, may bind directly in a non-covalent fashion to T cell receptors triggering a drug-specific immune reaction as described in the p-I concept (pharmacological interaction with immune receptors). This may explain non-IgE mediated reactions that occur within hours of first exposure. The reasons for the lack of reactivity for some hapten drugs or through the p-i model are not known [8]. Further understanding is needed that can influence future drug development and the preclinical prediction of which molecules may be likely to cause DHRs, leading to the development of germane molecules with similar pharmacological activity, but without inducing immune reactivity. Unfortunately, for most drugs, the allergenic determinants are unknown. Although genetic factors will be important, associated environmental factors may play major roles in the development of DHRs. For instance, the role of irritant molecules and viruses acting as co-factors or danger signals is intriguing and needs further analysis. Currently, DHRs are difficult to predict during the different drug development phases. To date, only a few drug allergenic determinants and mechanistic pathways have been identified. Translational multidisciplinary projects to understand the mechanisms of DHRs are required, and these may use epidemiology, experimental models, cell biology and molecular biology techniques, as well as biobanking. Limiting animal experimentation is desirable, and utilizing in silico data may provide accurate models.

## The international drug allergy symposium

With the above in mind, the editors of The Journal of Allergy and Clinical Immunology, The Journal of Allergy and Clinical Immunology: In Practice, and The World Allergy Organization Journal sponsored the International Drug Allergy Symposium. This symposium, which was held March 1, 2018 during the American Academy of Allergy, Asthma and Immunology (AAAAI)/World Allergy Organization (WAO) Joint Congress in Orlando, Florida, aimed at developing a series of international consensus documents. The controversies were grouped into five categories: 1) Penicillin and cephalosporin testing, 2) Radiographic contrast media, 3) In vitro testing, 4) Testing for delayed reactions, and 5) Drug allergy pathways. The information was presented by team leaders and discussed thoroughly in person and then evaluated by an expanded group of more than fifty experts around the world. Experts were asked to focus on *(1)* identifying the differences in standard management in the field, *(2)* providing evidence-based data supporting the different approaches, *(3)* developing potential expert opinion regarding best practices, and *(4)* designing future pragmatic research and clinical studies to evaluate outcomes. In order to guide and standardize the approach, we asked them to summarize the current knowledge on their topic in terms of *(1)* what we knew (already) and what we have (most recently) learned, *(2)* what is (still) controversial and/or done differently in different institutions or geographical areas (to show the relevant data addressing the alternate approaches), *(3)* what consensus recommendations can be made now? (single approach, “equal” alternatives, not enough information to make a recommendation), and *(4)* list the unmet needs (what is still not known, what requires further research, how to strategize global collaborations to answer the questions). The individual controversy and consensus statements are being concurrently published [9–13]. This article summarizes the key messages from those papers.

### Beta lactam testing

The group on penicillin and cephalosporin testing [9] identified the current most important concerns about beta lactam allergy, including the fact that mislabelling is widely spread across practices around the world, with the majority of patients claiming to be allergic being able to tolerate beta lactams after appropriate testing and challenge. The potential role of direct oral challenges without skin testing in children with low-risk clinical history is identified, and selective sensitivity to clavulanic acid is highlighted. Carbapenems and monobactams are described as safe alternatives for penicillin and cephalosporins allergic patients universally. There is overall agreement to avoid any further beta lactam exposure for patients with SCARs. Disagreements exit when choosing protocols and algorithms for delabelling and for in vitro testing for delayed reactions, which have not been validated. Standardizing the concentrations of major and minor determinants for penicillin and cephalosporins skin testing is needed. Recommendations are made for harmonizing protocols for diagnosis and testing and for the creation of large databases to provide outcomes. Additional research is needed in areas such as further understanding of risk factors, defining the natural history of allergy to penicillin and cephalosporins, and providing universal desensitization protocols is necessary.

### Radiographic contrast media

The group on radiographic contrast media (RCM) testing [10] classified worldwide phenotypes of ionic and non-ionic RCM reactions into acute and delayed, with non-severe delayed reactions accounting for the majority. Non-standardized approaches varied around the world regarding diagnostic testing, including skin testing and in vitro basophil activation test (BAT) and pre-medications regimes. The role of provocation challenges in negative skin testing patients with prior severe reactions to identify alternative RCM is controversial. Risks factors identified include atopy, asthma, female gender and especially prior reactions. Skin testing concentrations and premedication recommendations are provided but need validation, and algorithms are described which can be applied in multicentre studies to further understand outcomes.

### In vitro testing

The group on in vitro testing [11] reviewed diagnostic testing based on acute and delayed reactions phenotypes. While specific IgE, BAT testing and tryptase are of value for acute reactions, LTT, Elispot and cytokine measurements have been used for delayed reactions. Standardization of activation markers for BAT such as CD63 and CD203c and addressing its specificity for drug families is desirable. New biomarkers are described for severe delayed reactions, such as granulysin and granzyme B, and measurement of urinary mediators, such as histamine, prostaglandins and leukotriene, are of value in certain acute reactions. The specificity of in vitro testing is found to be high across the world, but the sensitivity is in general low, and it is recommended that these tests should be used to address specific clinical phenotypes and in association with in vivo testing. The availability and costs of these tests are limiting factors for widespread use as well as the lack of validation in different populations. Standardization of reagents and defining optimal drug concentrations and what constitutes significant increases above normal values are unmet needs.

### Testing for delayed reactions

The group on testing for delayed reactions [12] identified the need for standardized diagnostic and treatment approaches for delayed immunologically-mediated adverse drug reaction (IM-ADRs)/severe cutaneous adverse reactions (SCARs) which can be applied in multicenter studies. Because the cases are rare, there are difficulties in the early recognition, and the heterogeneity in phenotypes with non-classical and overlap symptoms may lead to misdiagnosis. A consensus committee is needed focusing on standardization of diagnostic criteria and treatment recommendations and procedures for the most common drugs and phenotypes. The use of in vivo tests, such as patch testing and delayed intradermal tests (IDT), in specific phenotypes is recommended. Use of in vitro testing available at the time of the acute reaction and at the time of evaluation is ideal, but there is no standardization of drug concentrations, vehicles, preparation and knowledge on stability of in vitro test solutions. Given the rarity of SCAR, large collaborative networks are needed to study the sensitivity, specificity and safety of IDT and patch testing as well as potential cross-reactive drugs and safe future drug choices. Genotype studies on at-risk populations are needed, and research on the immunopathogenesis of these reactions will help improve prevention, diagnosis and treatment.

### Drug allergy pathways

The group on drug allergy pathways [13] identified common factors associated with the growing mislabelling of allergic patients, including the increased antibiotic usage in developed countries, the emergence of antibiotic resistances, and the lack of current widely available tools or personnel to address patients with histories of adverse events at the time of antibiotics exposure. The morbidity associated with patients claiming to be antibiotic allergic has been surfacing in the last 10 years, and the need to identify true allergic patients among the majority of non-reactors has generated the need for pathways for in patient and outpatient use. Empiric pathways for the use of Penicillin and cephalosporins have been developed which are currently being validated and will need modifications based on the differences between countries regarding patients’ symptoms, exposures and prescription patterns. Patient phenotypes and endotypes are described as low, intermediate and high risk, and different approaches and recommendations are provided for each risk level. Because the outcomes of the pathways described can differ in different populations, modifications and adaptations will be necessary for each local application. Although universal pathways are desirable, local antibiotic usage will dictate their potential applications.
